# Comparison of the Polychromatic Image Quality of Two Refractive-Segmented and Two Diffractive Multifocal Intraocular Lenses

**DOI:** 10.3390/jcm12144678

**Published:** 2023-07-14

**Authors:** Luis Salvá, Scott García, Salvador García-Delpech, Anabel Martínez-Espert, Diego Montagud-Martínez, Vicente Ferrando

**Affiliations:** 1 Oftalmedic Salvà, 07013 Palma de Mallorca, Spain; lsalva@oftalmedic.com (L.S.); brscottgarcia@gmail.com (S.G.); 2 Clínica Aiken, Fundación Aiken, 46004 Valencia, Spain; 3 Departamento de Óptica, Optometría y CC de la Visión, Universitat de València, 46100 Valencia, Spain; anabel.martinez@uv.es (A.M.-E.); diemonma@upvnet.upv.es (D.M.-M.); 4 Centro de Tecnologías Físicas, Universitat Politècnica de València, 46022 Valencia, Spain; viferma1@upv.es

**Keywords:** intraocular lens, segmented intraocular lens, optical bench, longitudinal chromatic aberration, ray-tracing, extended depth of focus

## Abstract

Evaluating chromatic aberration for a multifocal intraocular lens (MIOL) in vitro is essential for studying its performance because it helps determine the most appropriate lens for each patient, enhancing surgical outcomes. While refractive MIOLs with angular power variation have shown positive clinical outcomes, studies of these MIOLs on optical benches primarily employed monochromatic green light, neglecting the impact of longitudinal chromatic aberration (LCA) on MIOL performance. To address this gap, we evaluated the through-focus modulation transfer function (TF-MTF) and the point spread function (PSF) of two refractive segmented extended depth of focus intraocular lenses (IOLs) (Femtis Comfort and Precizon Presbyopic), comparing the results with those obtained with two widely known diffractive multifocal intraocular lenses (AcrySof IQ ReSTOR and FineVision Pod F). Measurements of the TF-MTF were conducted using both monochromatic and polychromatic light in a customized optical bench. The refractive designs exhibited distinct haloes in the PSFs. When comparing the refractive and diffractive designs, opposite signs of LCA were observed at near foci. These findings emphasize the influence of the optical design of IOLs on their performance under polychromatic light, providing valuable information for vision care professionals when selecting the most suitable lens for each patient.

## 1. Introduction

The number of different models of multifocal intraocular lenses (MIOLs) for the correction of presbyopia has notably grown in the last few years, with over 50 different models in the market. Despite this extensive range, MIOLs can still be classified into two primary groups based on their optical principles: purely refractive and hybrid diffractive-refractive lenses. Hybrid diffractive-refractive lenses are MIOLs with a refractive base and addition, often referred to as “diffractive” in much of the literature [1,2]. Considering that we live in a polychromatic environment and both types of MIOLs exhibit a certain degree of chromatic aberration, understanding the differences in the polychromatic performance of MIOL models is important for better comprehending their clinical outcomes. Even if the chromatic behavior of MIOLs is primarily influenced by the refractive index and the Abbe number of the lens material [3,4], there is a significant influence of the lens design on the longitudinal chromatic aberration (LCA), which differs between the two groups of MIOLs. Although in hybrid designs, the diffractive profile can be designed to harness its chromatic behavior to some extent [5], in general, the addition generated by these MIOLs results in an LCA opposite to that produced by their refractive base. This is not the case with refractive MIOLs.

On the other hand, the correction of chromatic aberrations in the eye has been a subject of long-standing debate, and various proposals for LCA-correcting intraocular lenses have been put forth. While certain models have successfully achieved this correction, others have failed to produce a significant difference in LCA compared to phakic eyes [6].

From a clinical point of view, the lack of objective information on the chromatic properties of intraocular lenses (IOLs) often leaves ophthalmologists without the knowledge to inform their patients about their recommendations properly. This is especially true for the new refractive designs with angular power variation since the few studies carried out with polychromatic light have been done mainly with diffractive lenses [3,4,6,7,8]. The first refractive MIOL with an angular power distribution was the Lentis M-Plus (Oculentis, Arnhem, The Netherlands). This bifocal design has been recently adapted to the market demand for extended depth of focus lenses simply by reducing the additional power. The result is the Femtis Comfort (Oculentis, Arnhem, The Netherlands) model. Another example of refractive sectorial design in the market is the Precizon Presbyopic IOL (OPHTEC BV, Groningen, The Netherlands), advertised as the ‘continuous transitional focus intraocular lens’ IOL. In this design, the near and far refractive zones are spatially distributed in several sectors to provide an extended depth of focus effect independent of pupil size.

According to several studies, both Femtis Comfort and Precizon Presbyopic IOLs have shown good clinical results [9,10,11,12,13]. However, to our knowledge, the chromatic behavior of these two refractive models has never been objectively analyzed and compared. Therefore, this work aims to provide new evidence on the polychromatic behavior of extended depth of focus MIOLs with sectorial refractive zones on an optical bench.

The point spread function (PSF) and the through-focus modulation transfer function (TF-MTF) are the two descriptive metrics we used to assess the optical performance of these MIOLs. The modulation transfer function (MTF) is formally defined as the modulus of the Fourier transform of the PSF, resulting in a two-dimensional function. In lenses without symmetry around the optical axis, the MTF values vary along different meridians. Therefore, due to the lack of symmetry in the studied MIOLs, it is necessary to distinguish between tangential MTF and sagittal MTF. This differentiation is crucial to obtain a comprehensive evaluation of the optical performance of these MIOLs. In our case, the tangential MTF corresponds to the horizontal meridian, while the sagittal MTF corresponds to the vertical meridian. On the other hand, the TF-MTF describes the variation of the modulation transfer function of an optical system as the image plane is shifted across the focal region for a specific spatial frequency. The influence of pupil size on each model is also evaluated using the TF-MTF.

To place our results in context, two well-known models of apodized [14] diffractive lenses, one bifocal: AcrySof^®^ IQ ReSTOR^®^ (Alcon, Fort Worth, TX, USA) and one trifocal: FineVision Pod F (PhysIOL, Liege, Belgium), are also studied under identical experimental conditions.

## 2. Materials and Methods

### 2.1. Multifocal Intraocular Lenses

Four distinct models of MIOLs were examined and compared, comprising two purely refractive designs and two hybrid designs combining refractive and diffractive components. Table 1 summarizes the parameters of the IOLs employed in this study. Samples of IOLs of high power have been chosen to make the refractive effects more evident.

The Precizon Presbyopic IOL design is characterized by an aspherical segmented refractive optic on the anterior surface, with multiple zones divided into three concentric sectors. A narrow central sector is dedicated to distance correction, while the peripheral sectors present a clear bimodal distribution of distance and near correction, and this distribution changes along four segments in each sector. With this design, the manufacturers claim that this model offers a constant defocus between the two sharp focal points, delivering a good intermediate vision. Using four angular sectors, with alternate powers distributed in concentric rings, provides pupil size independence.

The Femtis Comfort IOL is a refractive lens with a rotationally asymmetric near-vision segment on one optic area. This IOL has a plate-haptic design with four flaps for capsulorhexis fixation. The distribution of the power zones of both refractive MIOLs will be shown in Section 3.1.

On the other hand, the two widely known diffractive IOLs were assessed for comparison: The AcrySof^®^ IQ ReSTOR^®^ SN60D3 + 4.0 D and the FineVision POD F. The first one is an apodized diffractive bifocal IOL [14]. The FineVision POD F is a trifocal IOL constructed with two bifocal apodized diffractive patterns, one for far-near vision and the other for far-intermediate vision.

### 2.2. Numerical Simulations

The performance of the two refractive MIOLs was first evaluated numerically using MTFs and PSFs metrics. To this end, we employed the Zemax OpticsStudio ray tracing software (v. 18.7, LLC, Kirkland, WA, USA), in which the experimental optical setup was programmed. The refractive profile of the Femtis Comfort IOL and the Precizon Presbyopic IOL was incorporated as a Grid Sag Surface on the front surface of each MIOL, with a radius of 21.4 mm. The posterior radius of each lens was chosen to get the power of MIOL employed in the experiments, and an aspheric surface was chosen as the back surface to achieve the spherical aberration as specified by the manufacturers. These values were r = −5.12 mm and Q = −2.35 for the Femtis Comfort; and r = −5.58 mm and Q = −2.30 for the Precizon Presbyopic.

Finally, images of a line of tumbling E optotype corresponding to 0.4 logMAR located at different vergences were simulated for both refractive MIOLs.

### 2.3. Optical Bench

The optical system used in our experiments was a custom-made imaging optical setup initially designed to meet the requirements of the ISO 11979-2 Standard [15,16]. The optical system was described in detail elsewhere [15] and was previously employed to evaluate different MIOLs designs [15,17,18]. It allows the measurement of the polychromatic TF-MTF of IOL since it can measure the light intensity distribution generated by a MIOL under test for an object located at different vergences from the eye model in which it was placed. In addition, in our system, the object can be either a pinhole to obtain the axial PSF; or a binary grating for obtaining the TF-MTF at a given spatial frequency.

**Figure 1:**
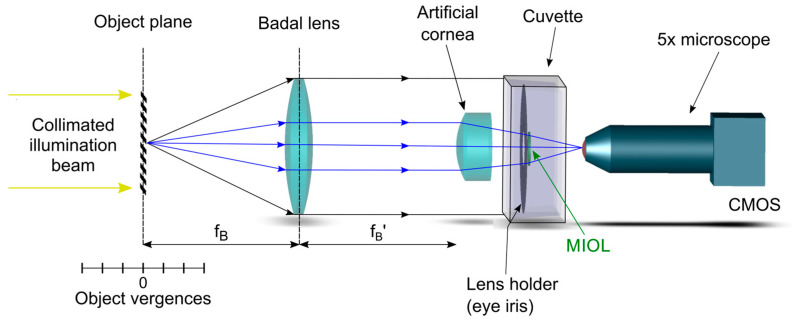
Optical bench setup. The object test can be located at different (positive or negative) vergences using the linear translation stage. The eye model is similar to the model 1 eye described in the ISO 11979-2 Norm, consisting of an artificial achromatic lens acting as an artificial cornea and a cuvette where the MIOL is immersed in a saline solution just behind the artificial iris. The aerial image behind the cuvette was recorded with a 5× microscope and a CMOS camera.

A schematic representation of the optical setup is reproduced in Figure 1.

The object is illuminated by a collimated beam from a white LED (Thorlabs MCWHL5), which can be filtered with different chromatic filters. In this experiment, we employed filters of 10 nm bandwidth, centred at 450 nm, 550 nm, and 650 nm (Thorlabs FB450-10; FB550-10; FB650-10). It was mounted on a stepping motorized translation stage (travel range 300 mm, accuracy: ±5 µm) to generate vergences ranging from −2 D to +6 D in steps of 0.04 D. Vergences were measured from the object focal plane of the Badal lens, an achromatic lens of focal length: 160 mm (see Figure 1).

The MIOL to be tested was placed sequentially in different holders with two pupil sizes; 3.0 mm and 4.5 mm. Then, it was immersed in a saline solution inside a cuvette with flat windows. This work uses an achromatic lens (Melles Griot LA034 27.8 D) as a model ISO 1 cornea [16]. As the cornea of the artificial eye was placed at the image focal plane of the Badal lens, the angle subtended by the test object, and consequently the spatial frequency assessed in the TF-MTF, was constant for all vergences [15]. Moreover, to isolate the polychromatic optical performance of the IOLs from the residual chromatic aberration generated by the optical elements in the optical bench, the system was calibrated by measuring the residual LCA (system without the IOL), and this result was subtracted from the experimental TF-MTF curves.

The camera and the attached microscope were mounted on an XYZ translation stage to adjust the image plane (the virtual retina) precisely. To obtain the PSFs generated by the MIOLs at different vergences, a 30 µm pinhole was employed as a test object. The TF-MTF at 50 cycles/mm was obtained with a periodic binary grating as a test object. From the calculation of the loss of contrast of the image of the grating registered by the CMOS camera, the MTF for each object vergence was automatically obtained [15]. The movements of the translation stage and the processing of the retinal images were controlled by custom software programmed in LabView. Due to its particular configuration, the system can measure the sagittal and tangential MTFs independently simply by rotating the orientation of the test grating 90 degrees. This aspect is essential due to the rotational asymmetry of the studied refractive lenses. Therefore, each measurement of the TF-MTF was obtained in both orientations, and the mean value of the sagittal and tangential counterparts was computed.

## 3. Results

### 3.1. Refractive Lenses Power Maps

**Figure 2:**
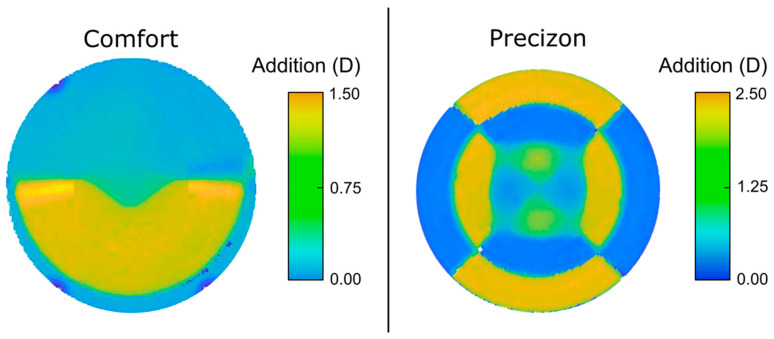
Relative power phase maps of the addition values obtained for the refractive segmented MIOLs normalized to their maximum value.

Figure 2 displays relative power phase maps of the segmented refractive MIOLs, classified as extended depth of focus. These maps were obtained using a commercial instrument, the NIMO TR1504 (Lambda-X, Nivelles, Belgium). To measure the range of powers accessible by the instrument, a negative lens with a power of −20 D was superimposed and centered with the MIOL in each measurement. Additionally, the base power of each lens was set to zero to obtain the distribution of the addition power on the surface of each model.

### 3.2. Numerical Results

**Figure 3:**
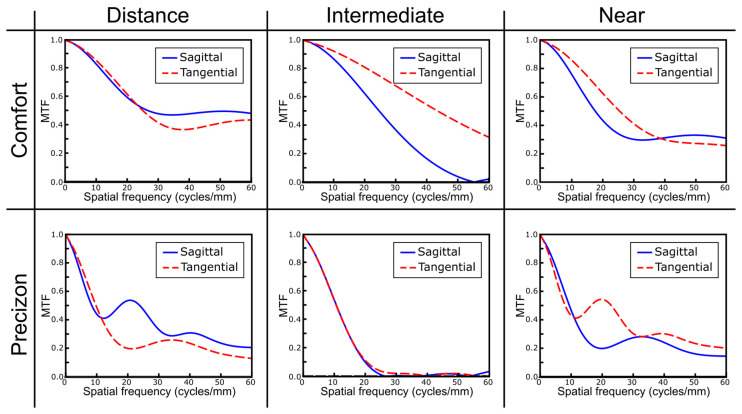
Tangential (dashed lines) and sagittal (continuous lines) modulation transfer functions (MTFs) computed up to 60 cycles/mm obtained at far, near and intermediate planes.

Figure 3 shows the sagittal and tangential MTFs of the two refractive lenses computed numerically at the two main foci (far and near) and an intermediate plane. Note that the difference in the sagittal and tangential polychromatic MTFs is particularly evident in the far and near foci of the Precizon Presbyopic MIOL, which look like those of toric IOL with the focal lines at the near and far foci.

**Figure 4:**
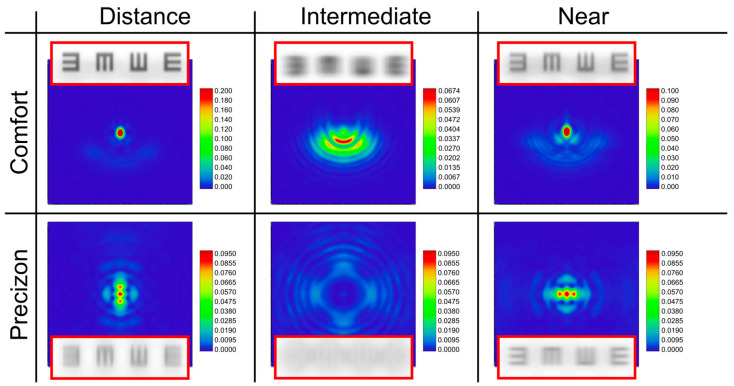
Point spread functions (PSFs) were obtained for a 3.0 mm pupil using Zemax software, corresponding to the same planes as shown in Figure 3. The color bars represent the relative intensity values. Each PSF is accompanied by a corresponding image simulation of a tumbling E optotype of 0.4 logMAR.

The asymmetries in the MTFs shown in Figure 3 suggest that the PSFs of both refractive IOLs should be different along the sagittal and tangential meridians. To verify this, we have calculated these PSFs at the same planes. The results are shown in Figure 4.

It can be seen how the asymmetric radial profiles produce a characteristic halo in their corresponding PSFs. The intensity levels in Figure 4 give valuable information for comparing the focusing performance of both refractive IOLs. The images of a tumbling E-line chart corresponding to a 0.4 logMAR visual acuity are displayed at the top and bottom of each PSF.

### 3.3. Experimental Results

**Figure 5:**
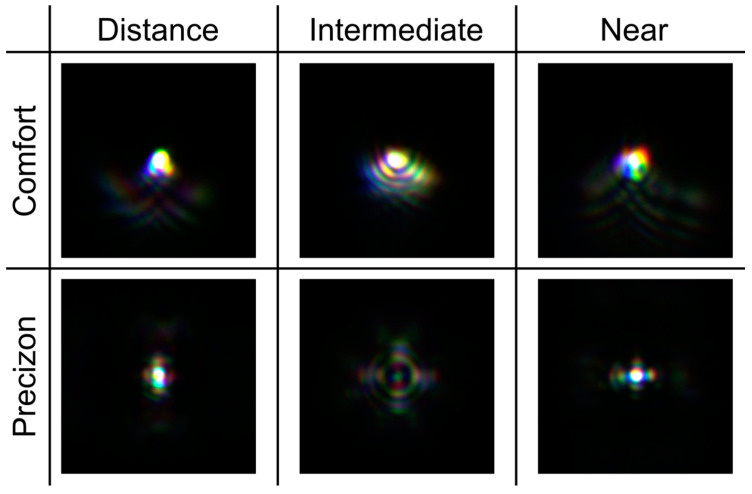
Experimental polychromatic PSFs for both refractive MIOLs.

Figure 5 compares the experimental polychromatic PSFs obtained for both MIOLs with segmented profiles obtained at the same planes as those computed numerically and represented in Figure 3 and Figure 4. In this figure, the camera integration time was adjusted to enable the visualization of the low-intensity halos surrounding the bright central peak. As a result, the brightest central spot in the far and near foci is intentionally saturated, using the same degree of saturation for each lens. A very good agreement can be observed between the experimental and numerical results (Figure 4 and Figure 5).

**Figure 6:**
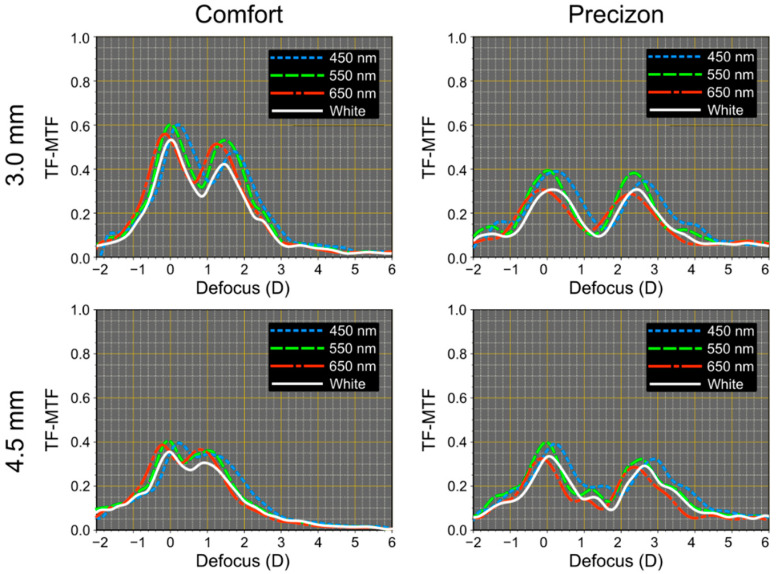
Experimental through focus modulation transfer functions (TF-MTFs) were obtained at 50 cycles/mm for the Femtis Comfort and Precizon Presbyopic MIOLs with pupil sizes 3.0 mm and 4.5 mm.

Figure 6 shows the experimental TF-MTF curves obtained, in the optical bench, for the refractive MIOLs at the spatial frequency of 50 cycles/mm, for pupil diameters of 3.0 mm and 4.5 mm using three different wavelengths (450 nm, 550 nm, and 650 nm) as well as white light. As expected, the order in which the peaks appear (red, green, and blue) in both the far and the near focus of the Precizon Presbyopic and Femtis Comfort MIOLs is consistent with their refractive nature.

The bifocal profile of both refractive lenses can be appreciated in this figure. The different values of the near add of each lens [18,19] are also evident. The Femtis Comfort IOL is pupil-dependent providing better TF-MTFs at the far focus for the small pupil. On the other hand, the more segmented design of Precizon Presbyopic IOL demonstrates its low-pupil dependency. However, the heights of the TF-MTF peaks of this IOL are lower than those of the Femtis Comfort, especially for 3.0 mm pupil. This result is also consistent with the intensities of the PSFs shown in Figure 4 and Figure 5.

**Figure 7:**
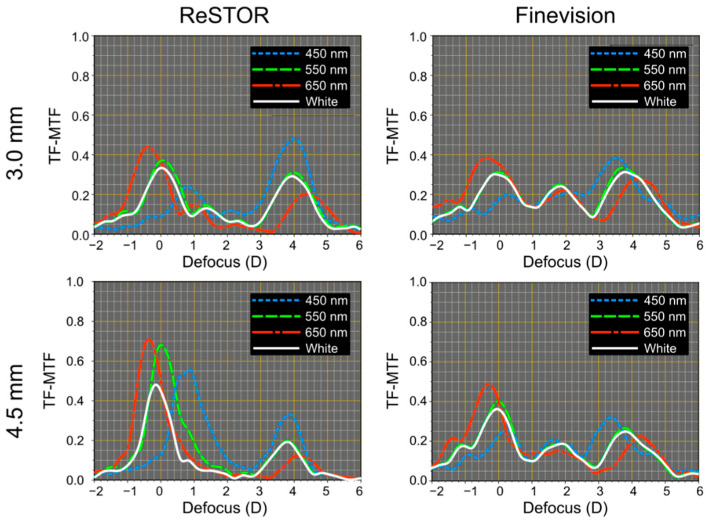
Experimental TF-MTFs at 50 cycles/mm were obtained for the ReSTOR and FineVision MIOLs with pupil sizes 3.0 mm and 4.5 mm.

To show the differences in chromatic responses between purely refractive segmented lenses and hybrid diffractive lenses, the polychromatic MTFs for two recognized MIOLs: the bifocal ReSTOR lens and the trifocal FineVision, have been obtained under the same experimental conditions. The results are shown in Figure 7. Note that in the far and near foci, the chromatic aberration is higher in both diffractive designs than in the refractive ones shown in Figure 6, especially in the near focus, where the sign of the LCA is opposite to the distance (refractive) foci. Besides, in the intermediate focus of the FineVision, the LCA is almost fully compensated. For the far focus, the difference between the diffractive and refractive models is more pronounced and can be attributed to the Abbe number of each lens material (see Table 1). It is important to note that the positions where the TF-MTF values obtained with white light were lower coincide with the ranges where the LCA is higher. Table 2 shows the values of the LCA (defined as the difference between the blue and red maxima of the TF-MTF at 50 cycles/mm) of the four MIOLs measured in this work.

## 4. Discussion

PSF and TF-MTF are two objective metrics that allow surgeons to differentiate MIOL designs. Since all MIOLs exhibit chromatic aberration, it is essential to understand their polychromatic performance for predicting and interpreting clinical outcomes. It has been shown that the optical quality of various EDOF IOLs differs when evaluated separately for different wavelengths [20]. The MTFs of EDOF IOLs are generally lower in polychromatic light than monochromatic light. Although differences exist between refractive and refractive designs, these effects are more pronounced at far vision than at intermediate distances. Therefore, an in vitro assessment of the optical performance of IOLs solely in monochromatic light is insufficient to predict their visual performance. A polychromatic study would better approximate the clinical (in vivo) situation. However, with few exceptions, in most of the literature MIOLs are still assessed only with green light, resulting in a general lack of information on this aspect. Furthermore, comparing the performance of different MIOL models becomes challenging when the published data lack comparability, especially when the experimental conditions in different studies are not the same [3,5,7,8].

In this work, new evidence of the polychromatic behavior of refractive sectorial MIOLs has been provided. It has been demonstrated that the asymmetric form of the refractive MIOLs produces characteristic halos in the PSFs, which are different from those produced by rotationally symmetric refractive MIOLs, for which the out-of-focus light is concentrated in symmetrical rings surrounding the focal points. To contextualize our work, the new results have been compared with those obtained under the same experimental conditions for other well-established diffractive MIOLs.

The Precizon Presbyopic model is promoted as an extended depth of focus lens because of the smooth transition between distance and near zones in the lens surface, which theoretically should be reflected the TF-MTF in an extended depth of focus between the two sharp focal foci. We found that on the optical bench, the TF-MTF for this lens is bifocal (see Figure 6). On the other hand, this lens is the least pupil-dependent IOL of this study.

The Femtis Comfort IOL has a design similar to another model from the same manufacturer (the Lentis M-Plus). Still, in this case, the addition was lowered to achieve an overlap between the far and near foci to obtain the extended depth of focus effect. This study confirms that this effect is also obtained on the optical bench, although the two differentiable foci are still present. Published studies reporting outcomes after insertion of the Femtis Comfort capsulorhexis-fixated IOL confirm that this IOL offers a very good functional range of vision and minimal unwanted visual phenomena [9,11,13]. Our results show that this goal could be achieved because although the glare is still visible in the experimental PSFs (see Figure 5), it has very low intensity (see the scales in Figure 4). This, in combination with the proximity of the foci, could justify that these effects may not have any clinically noticeable impact in most patients.

In comparing refractive and diffractive MIOLs, we found that refractive models have less LCA than diffractive ones (see Table 2). Furthermore, according to our expectations, we found that in the far foci of diffractive MIOLs (corresponding to the zeroth diffraction order), the LCA was positive and, therefore, directly linked to the refractive nature of the biomaterial. On the contrary, at near and intermediate foci, the values of the LCA were negative (see Table 2). In diffractive MIOLs, the distance focus exhibited higher LCA than refractive ones, and LCA remains within the same values for both pupil sizes. This finding coincides with that reported by Loicq et al. [3] for different models of diffractive multifocal IOLs. In previous work, Millán et al. [21] measured the LCA of different diffractive MIOLs, including the AcrySof^®^ IQ ReSTOR^®^ model we assessed in this study. Despite using a different metric (Energy Efficiency) and an experimental device that did not include a cornea in the artificial eye, the curves represented in that work for the distance (refractive) focus of this MIOL align with those obtained in the present study. However, contrary to the theoretical predictions, the authors also observed a positive LCA for the near focus. They argue that the LCA of the near focus caused by diffraction does not compensate for the LCA of refractive origin. The difference between their results and ours for the near focus could also be attributed to differences in the methodology and the different range of wavelengths evaluated. (450–650 nm vs. 455–625 nm).

This work was carried out with the limited sample of commercial lenses we had available at the time of the study. This fact could be recognized as one limitation of this study. However, we assumed these lenses had undergone quality control and were suitable for the study. Other optical bench studies have followed the same criteria (e.g., Refs. [3,5,22]). On the other hand, although the power of the studied lenses was not precisely the same, all are of high power (around 30 D), for which the tolerance limits in the manufacturing process are the highest: ±0.5 D [16,23].

Although the results in this study cannot be directly extrapolated to clinical expectation, mainly because the image quality of the eye under polychromatic light is influenced by other factors, especially monochromatic aberrations [23], the results we found are complementary to other studies of the polychromatic performance of MIOLs, since here two new refractive MIOLs models were assessed. Two diffractive IOLs were included in our research for the sake of comparison. The agreement between our results with those obtained in other studies for the same diffractive models [3,20] also adds reliability to the new results.

## 5. Conclusions

In conclusion, chromatic aberration significantly impacts the in vitro image quality of IOLs. Although with different responses, the studied EDOF IOLs demonstrated a clear potential to extend the visual range of patients with pseudophakia. The main differences in the optical performance of refractive sectorial MIOL lenses were observed in the polychromatic PSFs obtained in different image planes. The PSFs show that asymmetric designs are sensitive to the number and orientation of the segments.

We have observed that both the Precizon and Femtis Comfort lenses exhibit bifocal IOL characteristics in the TF-MTF curves. The Femtis Comfort has an addition of 1.5 D. In contrast, the Precizon lens has an addition of 2.5 D. Consequently, due to less addition (and more overlap between both foci), the intermediate distance vision of the Femtis Comfort lens could be better clinically compared to the Precizon lens.

Furthermore, we have demonstrated that the studied lenses exhibit varying degrees of sensitivity to different pupil sizes, indicating that their performance may not be equal under photopic or mesopic conditions. Specifically, the Femtis Comfort lens appears more pupil-dependent than the Precizon lens.

Additionally, we observed that the TF-MTF values obtained with white light, influenced by the longitudinal chromatic aberration (LCA), were consistently lower than those obtained with monochromatic light for all the examined IOLs.

These differences in performance among the studied designs can be helpful in surgeons when making decisions in cataract surgery, considering the particular visual needs of each patient.

## Figures and Tables

**Table 1:** For lenses included in this study, n is the material refractive index, d is the diameter of the optical zone of the multifocal intraocular lens (MIOL), An is the Abbe number of the MIOL material, Ad is the value of the near addition, and P is the MIOL base power. (Data provided by the suppliers).

MIOL (Type)	Material	n	d (mm)	An	Ad. (D)	**P (D)**
Precizon Presbyopic (Refractive)	Hydrophilic/hydrophobic acrylic material with ultraviolet filtering HEMA/EOEMA copolymer	1.46	6.0	47	2.50	28
Femtis Comfort (Refractive)	Copolymer, consisting of hydrophilic acrylates with hydrophobic surface	1.46	5.7	58	1.50	30
AcrySof^®^ IQ ReSTOR^®^ (Hybrid bifocal)	Acrylate/Methacrylate Copolymer	1.55	6.0	37	4.00	29
FineVision POD F (Hybrid trifocal)	Hydrophilic acrylic (25%)	1.46	6.0	58	1.75/ 3.50 *	33.5

* Intermediate/near addition.

**Table 2:** Longitudinal chromatic aberration (LCA) at a distance, intermediate and near foci of 3.0 mm and 4.5 mm pupil diameters of the refractive and diffractive MIOLs.

Pupil Diameter	MIOL	LCA (D)		
		Distance	Intermediate	Near
3.0 mm	Femtis Comfort	0.40	-	0.50
	Precizon Presbyopic	0.30	-	0.40
	AcrySof^®^ IQ ReSTOR^®^	1.20	-	−0.30
	FineVision POD F	0.55	−0.10	−0.50
4.5 mm	Femtis Comfort	0.40	-	0.20
	Precizon Presbyopic	0.40	-	0.65
	AcrySof^®^ IQ ReSTOR^®^	1.20	-	−0.20
	FineVision POD F	0.50	−0.10	−0.80

## Data Availability

The data supporting this study’s findings are available from the corresponding author, S.G.-D., upon reasonable request.
